# Galactosaemia − Should It Be Screened in Newborns?

**DOI:** 10.34763/devperiodmed.20182203.221224

**Published:** 2018-10-04

**Authors:** Annet M. Bosch

**Affiliations:** 1 Emma Children’s Hospital, Amsterdam UMC, University of Amsterdam, Department of Pediatric Metabolic Disorders, Amsterdam, the Netherlands

**Keywords:** galactose-1-phosphate uridyltransferase deficiency, classical galactosaemia, newborn screening

## Introduction

Classical galactosaemia (CG) is a disorder of galactose metabolism which results from a deficiency of galactose-1 phosphate uridyltransferase (GALT, EC 2.7.7.12) activity caused by mutations in the GALT gene (NM_000155.3). Patients have absent or barely detectable GALT enzyme activity and present in the first weeks of life with life threatening illness (feeding difficulties, liver failure, sepsis, cataract) after the ingestion of galactose from breast milk or infant formula. A lactose-free and galactose-restricted diet is life saving and the only available treatment at this time [1].

Newborn screening for CG by measuring GALT enzyme activity [2] was introduced in the 1960s with the expectation that early diagnosis and dietary treatment would prevent severe illness in the newborn period and that patients would have a normal outcome. However, since 1982 long-term complications have been reported in the literature. At this time it is clear that, in spite of an early diagnosis and immediate start of treatment, many CG patients suffer from long-term complications affecting their quality of life, such as impaired cognitive abilities, language and speech defects, neurological complications, and hypergonadotropic hypogonadism in females [3, 4, 5, 6, 7, 8, 9]. The most probable cause of these long-term complications is the persistent elevation of metabolites due to the endogenous production of galactose [10]. The fact that long-term complications are not prevented recurrently brings up the dilemma whether galactosaemia should be screened in newborns.

## Newborn screening for classical galactosaemia

Newborn screening has historically been implemented with consideration of the ten principles of Wilson and Junger [11]. Some of these principles are nowadays easily dealt with in most countries: the condition should be an important health problem; facilities for diagnosis and treatment should be available; case finding should be a continuing process. However, for CG newborn screening some other principles are still subject to discussion.

### There should be a suitable test or examination, which is acceptable to the population

Classical galactosaemia is mostly screened by GALT enzyme measurement, often combined with total galactose (TGAL) measurement. Screening will result in the identification of false positive (FP) cases, as has been demonstrated in several reports [12,13]. It is of utmost importance to minimize the number of FP referrals, as these have been demonstrated to cause anxiety, parentchild dysfunction and alterations in parental perception of the child’s health [14]. CG newborn screening in the Netherlands started in 2007 with a very high rate of FP referrals. Multiple adjustments since then in methods and cutoff values have now reduced the number of false positives [13]. A study in Sweden reported a decrease in the number of FP referrals to less than 0.01%, and a positive predictive value of 64% using a two-tier test strategy of GALT enzyme measurement followed by the measurement of total galactose [15]. An important limitation of combining GALT and TGAL measurements is that children on a low lactose or lactose-free diet (i.e. parenteral feeding or hypoallergenic formula) may not demonstrate elevated TGAL values and can be missed by newborn screening.

### There should be an accepted treatment for patients with a recognized disease

The only available treatment at this time is accepted and easily implemented: a life-long lactose-free and galactose restricted diet, which rapidly resolves the illness in the newborn period. It is advised to introduce this diet, which is in infants based on lactose free formula based on soy or a hydrolysate, as soon as the diagnosis is suspected, without awaiting results of diagnostic tests [16].

### The natural history should be adequately understood and there should be a recognizable latent or early symptomatic state.

Depending on the day of screening, most patients will be symptomatic and may already be hospitalized at the time of diagnosis by newborn screening [13]. However, early diagnosis and an immediate start of treatment significantly reduce morbidity and mortality. In Germany Schweitzer-Krantz (2003) demonstrated that early detection with newborn screening performed at day 5 prevented mortality in the first weeks of life, with death reported in 19/49 patients diagnosed before the implementation of newborn screening and 1/99 diagnosed after the start of newborn screening [17]. In Sweden only 4 CG patients diagnosed before the start of screening in 1967 survived, while since then only one patient has died [15]. Unfortunately, early diagnosis and treatment do not prevent long-term complications [17,18].

### There should be a policy on whom to treat as patients

Newborn screening often results in the detection of individuals with previously unreported phenotypes and genotypes, for whom the need for treatment and the potential outcomes are unclear. Indeed, in the Netherlands, after the start of CG newborn screening, 14% of the diagnosed patients had a previously unreported phenotype and genotype. These individuals did not demonstrate any symptoms at the time of diagnosis while still being exposed to galactose, and some demonstrated normal metabolite levels after the start of dietary treatment. The recent international guideline for CG recommends dietary treatment in all patients with GALT enzyme activity <10%, and states that there is insuffcient evidence whether patients with 10-15% of GALT enzyme activity need to be treated. In the Netherlands, currently all the patients with enzyme activity below 15% are treated. Future studies on the need for treatment and the outcome of these patients are warranted [13, 16].

### The cost of case finding should be economically balanced in relation to possible expenditure on medical care as a whole

A small number of studies evaluating cost benefits with different methods provide contradictory results [19, 20, 21].

## Discussion and conclusion

Currently newborn screening is performed in 9 European countries: Austria, Estonia (on a research basis), Germany, Greece, Italy, the Netherlands, Spain, Switzerland and Ireland. Two recent papers discussed the appropriateness of galactosaemia newborn screening. Varela Lema et al. (2016) concluded from their systematic review that there was insufficient evidence to establish the appropriateness of CG newborn screening, but that health benefIts could be expected if early diagnosis and treatment is achieved [22]. A Cochrane review published in 2017 concluded that it is not possible to draw conclusions based on randomized controlled studies, but that there are uncontrolled studies which support the efficacy of newborn screening for CG and that a number of reviews and economic analyses of non-trial literature suggest that screening is appropriate [23].

The striking effects of early diagnosis on the morbidity and mortality of patients in the first weeks of life strongly confirm the appropriateness of CG newborn screening, even when taking into consideration the fact that early diagnosis does not prevent long-term complications and the lack of insight into the cost-benefit ratios. However, with the implementation of CG newborn screening, It is of utmost importance to minimize the number of FP referrals and to educate the professionals involved in the treatment that if TGAL is one of the screening parameters newborns on low lactose diets may not be detected by the screening.

## From the Editor

In Poland newborn screening (NBS) for galactosaemia was introduced in the 1960s in parallel with NBS for phenylketonuria, homocystynuria, tyrosinaemia, histidinemia and maple syrup urine disease. A neonatal mass screening programme for the above inborn errors of metabolism was created, conducted and coordinated by the National Research Institute of Mother and Child (Instytut Matki i Dziecka, IMiD) in Warsaw – i.e. the Institute's team of biochemists and clinicians and their leader − Prof. Krystyna Bożkowa, who was then Head of the Department of Paediatrics and Director of the IMiD. She started cooperation with European centers and in 1963 with the USA (based on a bilateral agreement). Scientific collaboration between the IMiD and the National Institute of Child Health and Human Development in Bethesda, but also with centers in Baltimore, Boston, and Los Angeles, facilitated a very early introduction of NBS in Poland for some genetically determined metabolic diseases [1, 2].

As there were sparse data regarding either the diseases' incidence or the usefulness of the newly (at that time) elaborated screening tests (e.g. the Beutler and Baluda test), pilot studies were undertaken in order to check which conditions should be screened.

As far as galactosaemia is concerned, the qualitative assay of uridyltransferase galactose-1-phosphate (GALT) was measured in dry blood spot by the Beutler and Baluda test. This method detects either complete or partial (as in the Duarte variant) enzyme deficiency. In order to verify the quantitative enzyme assay, the isotope method described by Chojnacki and Sawicka was used [3].

In the period of March 1969 to July 1976 more than 500 thousand newborns were screened. The age of collecting dry blood spot was from four to seven days of life. A complete lack of GALT activity was detected in four children. All of them presented with clinical signs and symptoms of classical galactosaemia; two of them died in the neonatal period, still before diagnosis. In 31 cases the mean activity was around 20%. Among them five children showed clinical signs of galactosaemia. In 161 children GALT activity was about or above 50% [4].

Based on the above results, the incidence of galactosaemia in the Polish population was calculated as around 1:14 000 of live newborns.

Ten years later the Guthrie test was used for NBS for galactosaemia and galactose-1-phosphate was measured in red blood cells (by the method of Bożkowa) and also galactose in blood and urine (5). In all patients with zero activity of GALT, who were on a diet with lactose, the concentration of galactose-1-phosphate in red blood cells was higher than 25 mg/dL. As expected, an increased level of galactose was found in the blood and urine of all the patients with a typical clinical picture of galactosaemia. There were two asymptomatic patients, however, in whom only trace amounts of galactose were detected in blood and urine. All these patients were treated with a galactose-free diet.

During the period of 1976-1978 NBS for galactosaemia was replaced by selective screening, so now newborns with a family risk are investigated (on their first day of life) and also infants with clinical signs and symptoms. At the same time quantitative enzyme assay was performed in order to identify carriers of galactosaemia among those families in which the newborns presented with decreased or zero activity of GALT. Such management enabled the detection of new families at risk. Then, until 1984, while continuing selective screening, NBS for galactosaemia was reimplemented using both methods: the Beutler and Baluda and the Guthrie tests. The latter one could identify patients with a lack of either GALT or galactokinase activity. Among the over 155 thousand newborns investigated by both methods, one case of classical galactosaemia was detected - but at the time of diagnosis this child was already symptomatic [2].

Based on such observation as the fact that significantly more patients were diagnosed through selective screening (i.e. by clinical symptoms) than through NBS and the failure to prevent the occurrence of clinical signs and symptoms (including deaths) in those cases which had been detected by NBS, the decision to cease NBS for galactosaemia was undertaken (6).

It was only in the 1990s that controversies regarding NBS for galactosaemia appeared in the literature [7]. Currently it is still a subject of debate.

*Prof. nadzw. dr hab. n. med. Jolanta Sykut-Cegielska*
